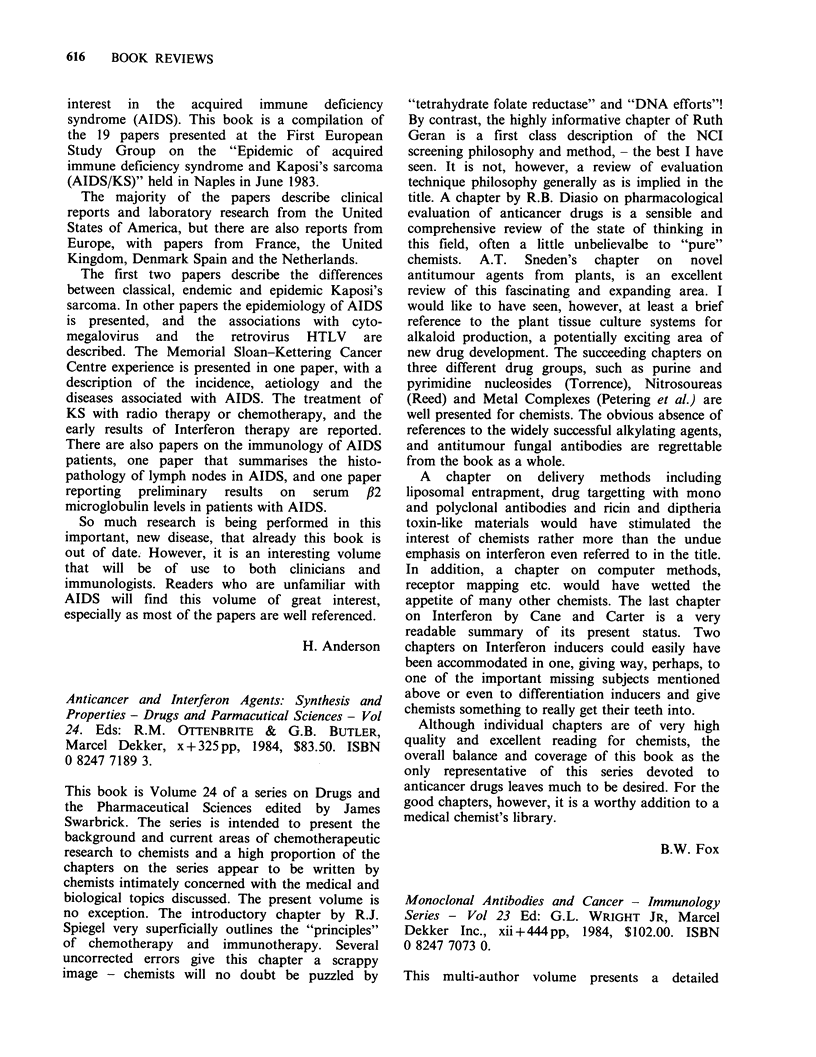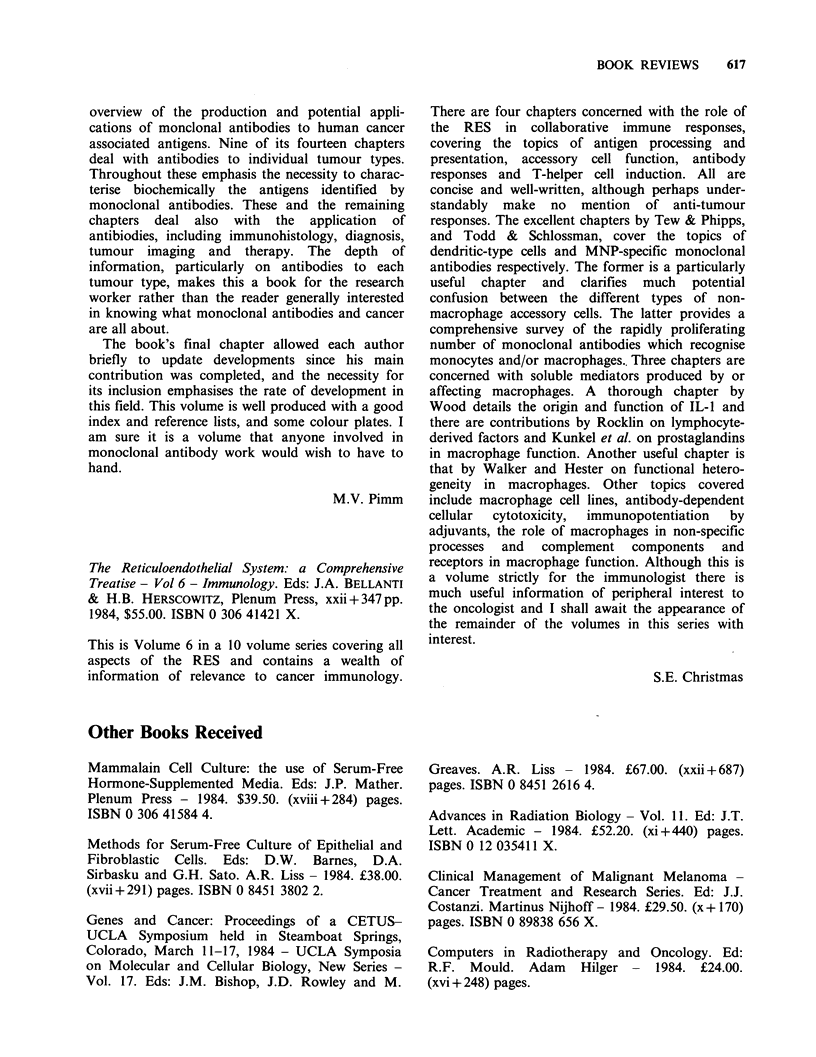# Monoclonal Antibodies and Cancer - Immunology Series - Vol. 23

**Published:** 1985-04

**Authors:** M.V. Pimm


*Monoclonal Antibodies and Cancer – Immunology Series – Vol 23* Ed: G.L. Wright Jr, Marcel Dekker Inc., xii+444pp, 1984, $102.00. ISBN 0 8247 7073 0.

This multi-author volume presents a detailed overview of the production and potential applications of monclonal antibodies to human cancer associated antigens. Nine of its fourteen chapters deal with antibodies to individual tumour types. Throughout these emphasis the necessity to characterise biochemically the antigens identified by monoclonal antibodies. These and the remaining chapters deal also with the application of antibiodies, including immunohistology, diagnosis, tumour imaging and therapy. The depth of information, particularly on antibodies to each tumour type, makes this a book for the research worker rather than the reader generally interested in knowing what monoclonal antibodies and cancer are all about.

The book's final chapter allowed each author briefly to update developments since his main contribution was completed, and the necessity for its inclusion emphasises the rate of development in this field. This volume is well produced with a good index and reference lists, and some colour plates. I am sure it is a volume that anyone involved in monoclonal antibody work would wish to have to hand.

M.V. Pimm